# Nitisinone desensitization protocol, case report of hereditary Tyrosinemia type 1 with successful treatment and outcomes

**DOI:** 10.1186/s13023-025-03666-8

**Published:** 2025-07-02

**Authors:** Michael Vallejo, Valentina Villarreal, María Alejandra Guardiola, William Bachiller, Malory Moreno, Pedro Vargas, Lyssethe Juliana López

**Affiliations:** Sociedad de Cirugía de Bogotá, Hospital de San José, Calle 10 # 18- 75, Bogotá, Colombia

**Keywords:** Tyrosinemia type 1, Newborn screening, Succinylacetone, Nitisinone (NTBC), Case report

## Abstract

The third known case in the country of Tyrosinemia type 1 is presented, a 10-month-old male infant who was referred to the emergency room due to hepatomegaly, compromised liver function, neurological deterioration, and abnormal urinary amino acids findings. Given the persistence of hepatic deterioration and focal hepatic lesions, a high clinical suspicion of Tyrosinemia type 1 was considered, and targeted treatment with Nitisinone and a restrictive diet was initiated, resulting in an appropriate clinical and tests response. However, there was an abrupt discontinuation of the medication without any medical indication, added to the fact that elevated succinylacetone levels were later reviewed, the main diagnosis was confirmed. The medication was continued, but the patient experienced adverse events related to it, requiring multiple dose adjustments provided by the medical team. Tyrosinemia type I is a rare disease and therefore difficult to diagnose, requiring extensive knowledge for timely and accurate treatment. Hence, the importance of acknowledging the management of these adverse events is crucial. The aim of this article is to present a rare clinical case and propose a desensitization protocol for Nitisinone for those cases that present adverse reactions to the medication.

## Introduction

Hereditary Tyrosinemia type 1 (HT-1), also known as hepatorenal Tyrosinemia, [1] is an inborn error of metabolism of autosomal recessive inheritance, caused by mutations in the fumarylacetoacetate hydrolase (FAH) gene located in the 15q23-q25 locus that encodes the fumarylacetoacetate hydrolase enzyme involved in tyrosine catabolism [2], which leads to the accumulation of toxic metabolites such as succinylacetone and maleylacetoacetate, [3] which causes different conditions such as severe liver cirrhosis, liver failure, neurological crises and even hepatocellular carcinoma [4].

It is the most serious type of the disorders spectrum associated with tyrosine metabolism. Its incidence in Central Europe is 1:125.000; however, this figure may vary according to the place where it is estimated. According to several pediatric sources its worldwide incidence is 1 in 100,000 live births, with the highest prevalence in Quebec (Canada), where its incidence is 1 per 16,000 individuals with predominance in the white race. [5] On the other hand, in Colombia, three cases have been reported, three cases have been reported, with the patient reported here being the only survivor to date based on what is found in the local literature [6, 7].

Clinical manifestations are broad, since the metabolic damage and the age of presentation depend on the level of enzyme deficiency. Even though an early diagnosis is done, with asymptomatic cases included, coupled with appropriate treatment can significantly improve the prognosis, [5] it is known that the severity of this disease in children has been made a big statement in medical literature, especially due to the potential development of hepatocellular carcinoma (HCC) and consequently, its fatality in the first years of life [8].

The purpose of this article is to characterize the appropriate management of the first Tyroseinemia type 1 patient in Colombia with success in treatment and therefore prognosis. Additionally, the intention is to propose a brand new protocol of NTBC to prevent its adverse events.

## Clinical case

A 10-month old male patient, from Bogotá (Colombia), without any personal nor family pathological history, with neonatal data of premature birth (34 weeks), who required management with oxygen therapy through a cephalic chamber and management in the neonatal intensive care unit (NICU) for 10 days due to suspicion of neonatal sepsis due to a history of maternal urinary tract infection (UTI); without relevant family or psychosocial history; who was referred to the genetics service because of an isolated hepatomegaly finding. The child was admitted to the emergency department in a stable condition, without any other associated symptoms. On physical examination, mucocutaneous paleness and hepatosplenomegaly were the only relevant findings. Laboratory tests were performed, revealing hyperbilirubinemia, cholestatic pattern, slightly elevated transaminase levels, and prolonged clotting times.

Plasma and vitamin K transfusion were initiated and studies were carried out for hepatotropic viruses (cytomegalovirus, Epstein Barr, hepatitis B surface antibody and hepatitis C), which were negative. In addition, myelogram was requested, which yielded a normal result. During hospital stay, he presented progressive clinical deterioration among neurological compromise performed by altered state of consciousness and respiratory state, whence he was transferred to the pediatric intensive care unit. The patient required ventilatory support (high-frequency ventilation) and developed altered urinary amino acids (elevation of serine, glutamine, glycine, phenylalanine, alpha-butyric, cysteine and tyrosine), altered lactate/pyruvate ratio, elevated ammonium and very high alpha-fetoprotein. Consequently, Lactulose, Rifaximin and hemodiafiltration were initiated without success, as clinical deterioration persisted due to acute respiratory distress syndrome (ARDS), multilobar pneumonia and hemodynamic compromise. Inotropic support and broad-spectrum antibiotics were administered, and hepatopulmonary syndrome was ruled out.

Subsequently, the patient was referred to another institution for assessment by the transplantation program, which considered repeating amino acid in urine tests due to a possible diagnosis of metabolic disease; an increase in the same amino acids previously reported was found, as well as a finding in abdominal ultrasound with multifocal micro liver lesions. Even though a few differential diagnoses were considered such as galactosemia, Wilson’s disease and hereditary fructose intolerance; there was high suspicion of Tyroseinemia type 1, therefore Nitisinone management was initiated with a favorable clinical and paraclinical response, which supported the diagnosis. Acknowledging, the patient wasn’t treated with any medication prior to that time.

**Fig. 1:**
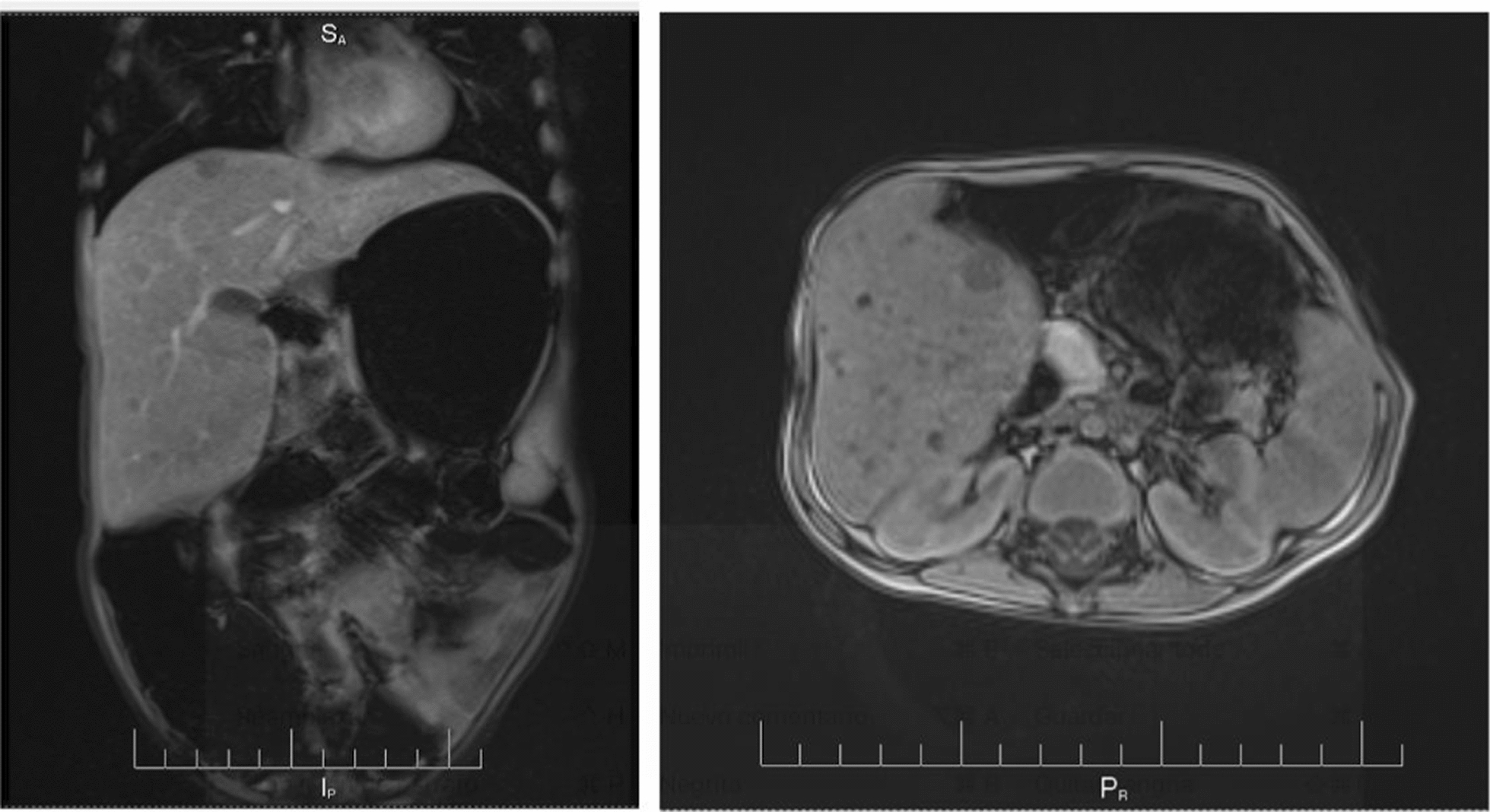
Abdominal MRI after NTBC suspension. Hepatomegaly with solid focal lesions, the largest of which was 11 mm and involved both lobes

**Fig. 2:**
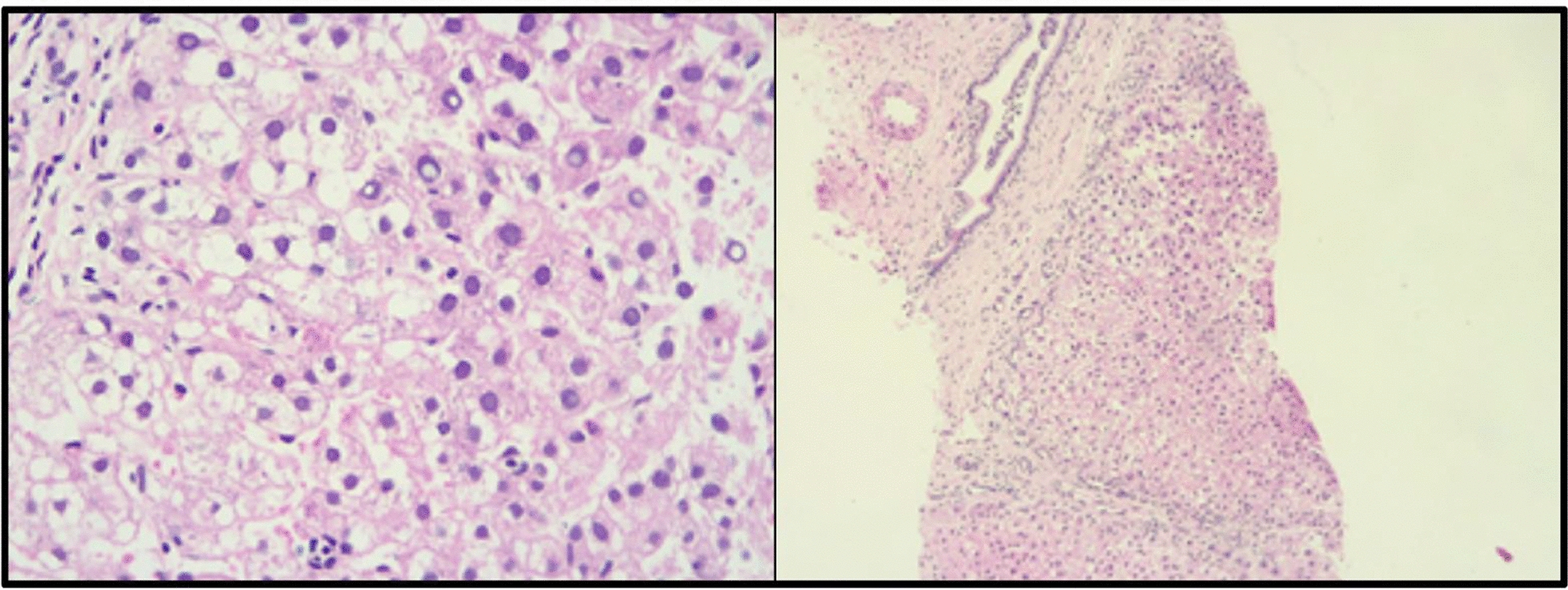
Liver biopsy that shows low representation of the portal triad with focal disruption of the architecture without presence of dilatacion of tracts or formation of regenerative nodules

However, the mother discontinued treatment with Nitisinone and the restrictive diet. Six months later, she attended the emergency department with the patient due to prolonged and frequent liquid stools and emetic episodes, which did not improve with outpatient measures. The physical examination found the patient in regular condition, with drowsiness, type-III DHT, smell of aged wood was identified in moments of crisis, altered liver function and MRI of abdomen with multiple hepatic nodules findings (Fig. 1). Given a high suspicion of Tyroseinemia type 1, the child was assessed by a multidisciplinary team (pediatrics, clinical genetics and nutrition), paraclinical tests were taken and a restrictive diet of phenylalanine and tyrosine was initiated, in addition to treatment with Maltodextrin and Nitisinone; the patient had a significant improvement. The report of the laboratory tests requested during critical conditions showed increased hydroxyphenylacetic acid, elevated 4-hydroxyphenylpyruvic excretion and succinylacetone; the latter confirmed the diagnosis of Tyroseinemia type 1. Considering the finding of hepatic lesions, a hepatic biopsy was requested to rule out hepatocarcinoma (Fig. 2).

**Fig. 3:**
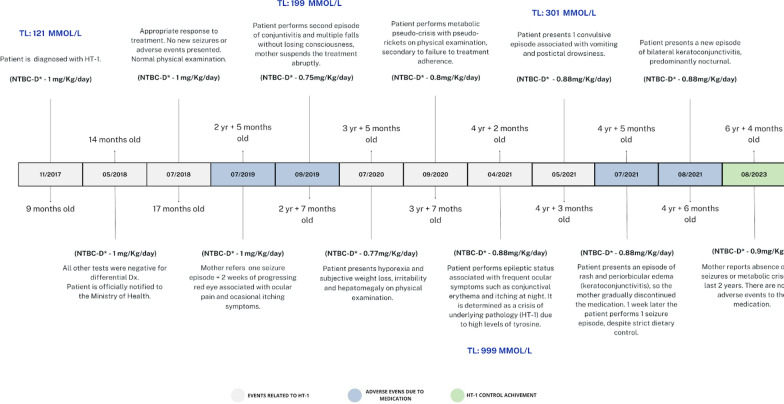
Timelapse of adverse events related to Nitisinone through the years since the diagnosis

Once the official diagnosis is established and the respective treatment is initiated, the patient has an appropriate response with normal physical examination on every next medical control visit. However, approximately two years later after being diagnosed, patient started performing multiple seizures and noticeable adverse reactions such as eye nuisance based on eye pain and keratoconjunctivitis, besides the numerous metabolic crises and rickets caused by the underlying pathology (HT-1); which led the mother of the child to abruptly withdraw the medication many times. This causes the medical professionals to modify the dose of Nitisinone several times to finally see the patient 4 years later at the age of 6 years, in a stable health and personal status, without any new adverse events reported for the last 2 years. Therefore, there is the expectation of having changed the forecasts towards an improved outlook. The Time-lapse of events performed by the patient related to the medication and progress of the illness is explained in (Fig. 3).

### Treatment protocol

The medication used to treat this condition is known as Nitisinone tablets of 2 mg. The established dosage ranges from 0.5 to 2 mg/kg/day during his treatment divided into two daily doses [1]. Adverse events occurred at a dose of 1 mg/kg/day. Prompting a gradual dose reduction according to our protocol to prevent NTBC intoxication due to tyrosine increase was established (Table 1). Following the completion of the seven-day desensitization protocol, the weekly dose was gradually increased, starting at 0.5 mg/kg divided into two daily doses, with a weekly dose increase ensuring optimal plasmatic tyrosine levels (200- 600 μM/L) [1] (Table 2). This resulted in an established final dose of 0.9 mg/kg divided into two doses.

**Table 1:** Nitisinone desensitization protocol

	Day 1	Day 2	Day 3	Day 4	Day 5	Day 6	Day 7
AM	1 Pill	½ Pill	½ Pill	X	X	X	X
PM	½ Pill	½ Pill	X	X	X	X	X

AM = morning dose, PM = night dose, X = not receiving treatment

**Table 2:** Instauration dosage of Nitisinone after desensitization protocol

Weeks	Dose
1	0.5 mg/Kg/day
2	0.6 mg/Kg/day
3	0.7 mg/Kg/day
4	0.9 mg/Kg/day

## Discussion

As mentioned before, Tyroseinemia is a rare disease, with variable incidence due to different factors, (Table 3) including initial non-specific symptomatology, lack of knowledge about the disease and poor diagnosis. In Latin America, there is no precise data on estimated incidences until the present [1].

**Table 3:** Risk factors for HT-1

Modifiable risk factors	Non-modifiable risk factors
Late diagnosis	Parents who carry the gene that causes the disease
Low economic resources	White ethnicity
Lack of accessibility of Nitisinone or low phenylalanine formula	

Adapted from: (3)

As reported by A. Dass [9], very few countries currently have policies for newborn screening with tandem mass spectroscopy; therefore, diagnosis is still made late, when clinical manifestations are very aggressive or even lethal. For the medical staff, it is important to recognize the symptoms, proceed with a biochemical diagnosis and conduct timely pharmacological interventions to avoid fatal consequences [9].

**Fig. 4:**
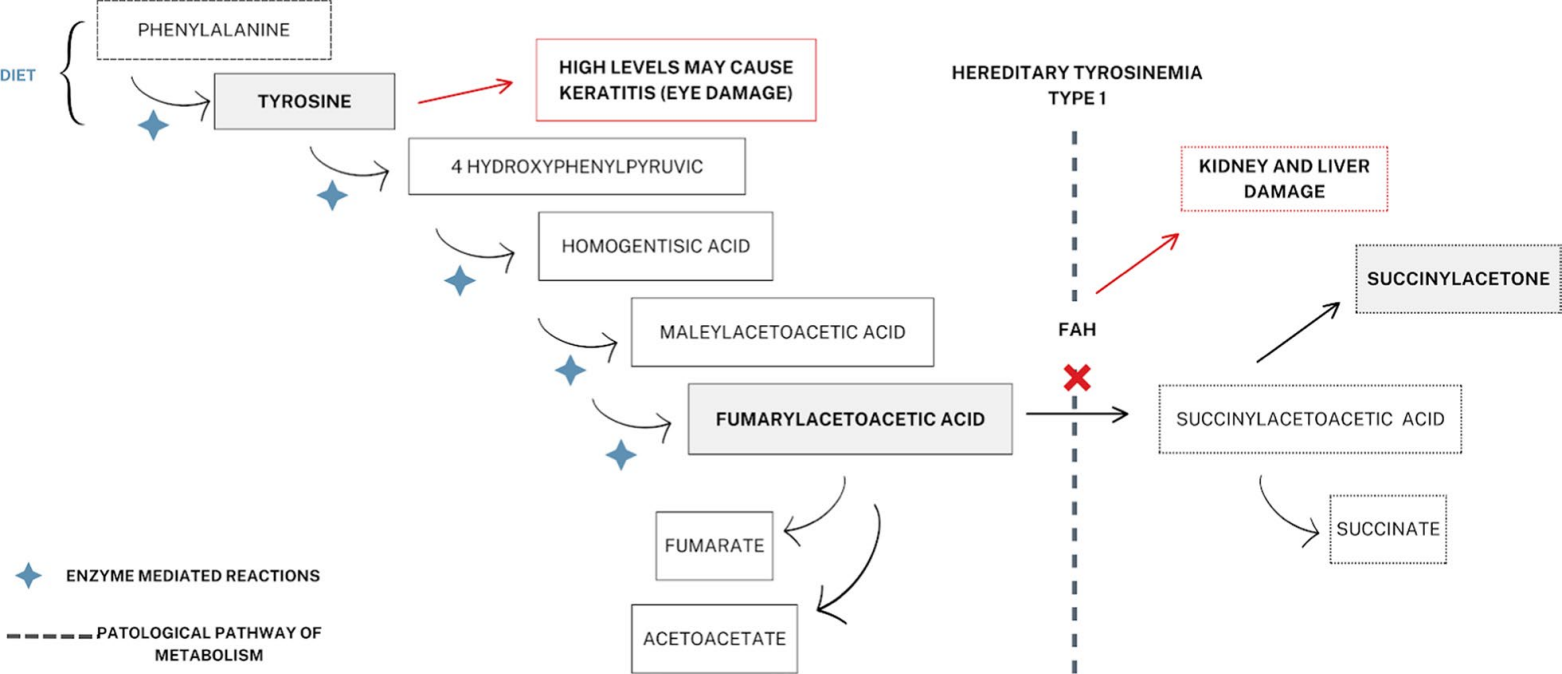
Tyrosine metabolism and side effects of FAH deficiency on HT-1

The biochemical knowledge of the disease is highly relevant to understand its clinical implications, as well as to direct the medical treatment (Fig. 4). Fumarylacetoacetate hydrolase is the enzyme responsible for the conversion of fumarylacetoacetate acid into fumaric acid and acetoacetic acid, the latest products of tyrosine catabolism. When this enzyme is deficient, fumarylacetoacetate is transformed into the toxic metabolites succinylacetone (SAA), fumarylacetoacetate (FAA) and maleylacetoacetate (MAA). SAA causes the renal tubular dysfunction that leads to Fanconi syndrome of variable expression; it also inhibits the delta-aminolevulinic acid (ALA), which is the enzyme that mediates the formation of porphyrins, which generates a decrease of heme iron. On the other hand, MAA and FAA act as alkylating agents able to bind to the DNA, promoting mutagenesis and apoptosis in the liver mainly [10, 11].

The clinical manifestations of type I Tyroseinemia are highly variable and an affected individual can present at any time from the neonatal period to adulthood. There is considerable variability in presentation even among members of the same family. It is described that this disease clinically manifests in three different scenarios based on age of symptom onset: an acute form shown before 6 months with acute liver failure, a subacute form between 6 months and 1 year presented mainly with liver disease, growth failure and rickets; and finally a chronic type after the first year of life with various complications such as cardiomyopathy, and/or a porphyria-like syndrome. Treatment with Nitisinone in the past 25 years has significantly changed its course [12–15].

Since the detection of this disease in our environment is done selectively, it is important to acknowledge the main clinical scenarios by each system. According to the reports [10, 11], the following manifestations may be observed (Table 4) [16].

**Table 4:** Main clinical and biochemical manifestations of HT-1 (14, 15, 16)

System	Symptoms	
	More common	Less common
Liver and gastrointestinal	Abdominal pain, hepatomegaly, splenomegaly, cirrhosis, liver dysfunction, ascites	Diarrhea, melena, fever, ileus, portal hypertension, hepatocellular carcinoma, esophageal varices
Kidney	Nephromegaly, rickets, tubulopathy	Fanconi syndrome, kidney failure
Nervous system	Irritability, seizures	Encephalopathy, change in mental status, head and trunk hyperextension
Others	Malnutrition, dehydration, cardiomyopathy, porphyria-like syndrome, etc	
Biochemical findings		
Anemia, hypoglycemia, pathological clotting times, thrombocytopenia, amino aciduria; high alkaline phosphatase, transaminases and bilirubin; acidosis, etc		

The average age of diagnosis due to clinical suspicion is directly proportional to the degree of severity and mortality, that is, around 15.5 months, although in places where newborn screening is mandatory, the average age of diagnosis is 0.55 months, which favors timely treatment and reduces complications [11].

Worldwide, the recommendation on the active and early search for the disease includes newborn screening, which indicates taking tests for succinylacetone levels to all newborns. The measurement of tyrosine levels as a primary marker of neonatal screening is not recommended, as some patients may present transient hypertyrosinemia at birth or some patients with Tyroseinemia type 1 may have normal tyrosine levels at birth [1].

In infants with clinical suspicion in whom newborn screening has not been performed, taking blood or urine levels of succinylacetone should be considered immediately as a confirmatory test. If positive, treatment with Nitisinone should be initiated and a genetic test for detection of the mutation should be performed later. Additional tests recommended to monitor liver function include clotting times and alpha-fetoprotein for liver damage, although results are expected to be normal in newborns [11, 17].

Immediately after the diagnosis of Tyroseinemia type 1, specific treatment and nutritional therapy should be initiated, acknowledging that there are places where blood or urine measurement of succinyl acetone cannot be done, but there is a high clinical suspicion that requires initiating the specific treatment [16, 18].

The treatment of HT-1 has evolved considerably in recent decades. Medical resources show that before the 1980s, the only proposed treatment was a diet restricted in both phenylalanine and tyrosine, in the hope of achieving successful therapy, following the success of early dietary treatment for phenylketonuria. [19] Unfortunately, when only treated with a phenylalanine/tyrosine restricted diet, the outcome was extremely poor. [20] Nowadays, the management of Tyroseinemia type 1 mainly focuses on reducing toxic metabolites (maleilacetoacetic acid, fumarylacetoacetic acid and succinylacetone) and is carried out by adding one more step [8, 21]:

1. Administration of Nitisinone to block tyrosine degradation by inhibiting 4-hydroxyphenylpyruvate dioxygenase, enzyme responsible for transforming phenylpyruvic acid into homogentisic acid. In this way, the production of succinylacetone and other toxic metabolites is not allowed, which is the mainstay of the treatment. The suggested dose is 1–2 mg/kg/day, twice a day, but similar results have been achieved when administered once a day, which could favor adherence to the treatment. Patients in crisis often show rapid improvement with this treatment, although the accumulation of tyrosine occurs as a side effect [21]. There has also been a decrease in the incidence of major complications such as hepatocarcinoma, showing that patients who start this treatment after one year of life have 13 times more risk of developing a hepatocarcinoma than those who started treatment in the neonatal period. The risk of liver cirrhosis, renal tubular dysfunction and rickets increases 40, 4.3, 19 times, respectively [1, 21].
2. With the exception of mild cases of PAH deficiency, reducing blood PHE levels through dietary modifications aimed at restricting PHE (and protein) intake leads to insufficient protein supply for normal growth and health. The cornerstone of treatment for PAH deficiency remains dietary therapy, [22] involving reduced consumption of natural protein and its substitution with a protein source (such as an amino acid mixture) lacking both components. [23] Consequently, patients in this scenario are prescribed a strict diet supplemented with a PKU medical formula containing a blend of phenylalanine-free amino acids, akin to those utilized for individuals with phenylketonuria.

Treatment follow-up is performed based on plasma tyrosine levels, since the therapeutic objective is to achieve tyrosine < 400 μM, and negative succinylacetone [4, 21].

After diagnosis and immediate initiation of treatment, clinical and laboratory test runs follow-up is essential. The American College of Medical Genetics and Genomics recommends strict follow-up as described in Table 5.

**Table 5:** Follow-up recommendations in patients with Tyrosinemia type 1

Follow-up	B. Tx:	12 months of age		12 months- 5 years			> 5 years
		Monthly	3 m	3 m	6 m	Annual	
**Serum SAA**	**x**	**X**		**X**			**Every/6 m**
Urine SAA **	**x**	**X**		**X**			**Every /6 m**
**Plasma a.a**	**x**	**X**		**X**			**Every /6 m**
*Complete blood count*	*x*		*X*			*X*	*Every/year*
*AFP*	*x*	*X*	*X*		*X*		*Every /6 m*
*PT and PTT*	*x*	*X*	*X*			*X*	*Every/year*
*Transaminases*	*x*	*x*	*X*				*Every/year*
*CT, MRI or abdomen ultrasound*	*x*						*Every/year*
*Renal ultrasound*	*x*						
*Kidney function tests*	*x*					*X*	*Every/year*
*Ca and P*	*x*					*X*	*Every/year*
*Urinalysis*	*x*						
*Nutritional follow-up tests*	*x*					*X*	
*Developmental assessment*	*x*	*X*				*X*	
*Neuropsychological follow-up*	*X*						
*Ophthalmologic follow-up*							*With symptoms*

Taken from the American College of Medical Genetics and Genomics.

Italics Tyrosinemia type 1 Markers

Bold Follow-up tests

B. Tx: beginning of treatment; SAA: succinylacetone; * in case of serum SAA is not available; a. a: amino acids; AFT: alpha fetoprotein; PT: prothrombin time; PTT: partial thromboplastin time; Ca: calcium; P: phosphorus

The most important complications are described to include acute liver failure, liver cirrhosis and a high prevalence of hepatocarcinoma, complications that require liver transplantation and may lead to death [24], which in this case-scenario are not yet established in the patient in question. A comprehensive follow-up should consider studying the siblings of the patient to discard or confirm this entity, in order to provide a timely treatment [16].

## Addressing HT-1: crucial strategies for coping with adverse events successfully

Nitisinone is a medication known to be used as an adjunct to dietary restrictions for the treatment of hereditary Tyroseinemia type 1 (HT-1), which causes intolerance to foods containing tyrosine. [25] Its main action mechanism is to competitively inhibit 4-hydroxyphenylpyruvate dioxygenase, an enzyme that intervenes in the tyrosine catabolic pathway, preventing the accumulation of catabolic intermediates (maleylacetoacetate and fumarylacetoacetate). (Fig. 4).

It has been described in the medical literature that the most common adverse reactions to Nitisinone are blood disorders (e.g., thrombocytopenia, leukopenia, etc.), eye nuisance (e.g., eye pain, conjunctivitis, red eye, etc.) and skin changes (e.g., rash, generalized itching, etc.). [26] These are mainly related to high levels of tyrosine and are reversible when its levels are reduced by a strict compliance diet. [9].

In this case, the main adverse event related to the medication is quite noticeable (Fig. 3) since the patient underwent periodic ophthalmological check-ups with a slit lamp, acknowledging physical examination never revealed tyrosine deposits, only signs of recurrent keratoconjunctivitis. Considering that those multiple seizure episodes, rickets and even visceromegaly manifested by the child, are symptoms and clinical signs described as frequent in the underlying disease and not as possible side effects of this drug.

The lack of neonatal screening policies leads to a late diagnosis, associated with decreased quality of life and worse prognosis, which is why it is recommended to perform Succinyl Acetone levels in blood or urine, this test works as screening in newborns and confirmatory test in children with clinical suspicion.

A positive result indicates the start of treatment with Nitisinone, a medication that blocks the action of 4-hydroxyphenylpyruvic acid, which does not allow the formation of toxic metabolites, reduces comorbidities, improves prognosis, reduces mortality, and allows normal development and quality of life for them. Treatment monitoring is carried out with plasma tyrosine levels, in which the therapeutic objective is to achieve tyrosine < 400 M, and a negative succinylacetone [4].

This disease has been the reason for experimentation with gene therapy in models. animals that seek to correct the mutation mediated by CRISPR/Cas9, which showed the no progression of liver cirrhosis, regeneration of up to 95% of hepatocytes which suggests that the addition of the genome could lead to being a valuable and safe therapy for this disease. However, this study in question is in phase II of experimentation [8].

In terms of controlling Nitisinone’s adverse effects a desensitization protocol was created based on this child’s specific case in order to continue using the medication which controlled the disease successfully, hence, its prognosis, modifying the dosage on multiple occasions considering the clinical condition and progress of laboratory tests. This protocol strategy is suitable and replicable in patients who present adverse reactions to this medication and require its use to avoid future complications of the disease.

## Conclusions

Tyrosinemia type 1 is a genetic disease, with high mortality rates secondary to the complications derived from the accumulation of toxic metabolites. It is characterized by bizarre clinical manifestations, which may also occur when there is already involvement of other organs such as liver (hepatocarcinoma being the most prominent) and kidney (Fanconi syndrome), which generates a late diagnosis associated with reduced quality of life and worse prognosis.

Newborn screening using levels of succinylacetone in blood or urine is recommended, and in countries where such screening is not available, diagnosis is made based on clinical suspicion. Since the gold standard test is the measurement of succinylacetone, if the result is positive, treatment with Nitisinone should be initiated; this drug has changed the history of patients with this disease, as its mechanism of action improves prognosis and decreases morbidity and mortality, allowing for a normal development and quality of life for these patients.

This disease has been the subject of experimentation with gene therapy in rat models that seek to correct the mutation mediated by CRISPR/Cas9. It has shown no progression of hepatic cirrhosis and the regeneration up to 95% of hepatocytes, which suggests that the addition of the genome could be a valuable and safe therapy for this disease. However, this study is in phase II trials. [4]

Ultimately, effective management of Nitisinone-associated adverse effects is crucial to ensure treatment safety and compliance in patients with HT-1. Early identification, continuous monitoring and timely intervention are essential to minimize the risks and maximize the therapeutic benefits of this drug, avoiding failures in medication adherence. The appliance of this protocol is an excellent choice that can be implemented in several populations, particularly in countries that do not have neonatal screening for metabolic disorders. It has proven to be efficient and safe for the initiation and continuation of the medication, thereby providing controlled treatment for patients with Tyroseinemia type 1.

## Data Availability

Not applicable.

## Abbreviations

HT-1: Tyrosinemia type 1
NTBC: Nitisinone
FAH: Fumarylacetoacetate hydrolase gen
HCC: Hepatocellular carcinoma
NICU: Neonatal intensive care unit
UTI: Urinary tract infection
MRI: Magnetic resonance imaging
ARDS: Acute respiratory distress syndrome
DHT: Dehydration
SAA: Succinylacetone
FAA: Fumarylacetoacetate
MAA: Maleylacetoacetate
ALA: Delta-aminolevulinic acid
DNA: Deoxyribonucleic acid
PAH: Pulmonary artery hypertension
PHE: Phenylalanine
PKU: Phenylketonuria
CRISPR: Clustered regularly interspaced palindromic repeats

## References

[1] Chinsky JM, Singh R, Ficicioglu C, van Karnebeek CDM, Grompe M, Mitchell G, Waisbren SE, Gucsavas-Calikoglu M, Wasserstein MP, Coakley K, Scott CR. Diagnosis and treatment of tyrosinemia type I: a US and Canadian consensus group review and recommendations. Genet Med. 2017. 10.1038/gim.2017.101. 28771246 10.1038/gim.2017.101 PMC5729346

[2] Bahador A, Dehghani SM, Geramizadeh B, Nikeghbalian S, Bahador M, Malekhosseini SA, Kazemi K, Salahi H. Liver transplant for children with hepatocellular carcinoma and hereditary Tyrosinemia type 1. Exp Clin Transplant. 2015;13(4):329–32. 10.6002/ect.2013.0158. 24679101 10.6002/ect.2013.0158

[3] Toapanta Ortiz A. Tirosinemia Tipo I. http://dspace.espoch.edu.ec/bitstream/123456789/9050/1/94T00352.pdf, 2018 [accessed 15 April 2024].

[4] Shao Y, Wang L, Guo N, Wang S, Yang L, Li Y, Wang M, Yin S, Han H, Zeng L, Zhang L, Hui L, Ding Q, Zhang J, Geng H, Liu M, Li D. Cas9-nickase-mediated genome editing corrects hereditary tyrosinemia in rats. J Biol Chem. 2019;293(18):6883–92. 10.1074/jbc.RA117.000347. 10.1074/jbc.RA117.000347 PMC5936814 29507093

[5] Fuentes-Cortés I, Pacheco-Suárez B, Charón-Savón D. Implementación de una metodología para la detección de marcadores bioquímicos en la tirosinemia tipo 1. Finlay. 2023; 13(4): 425–34. https://revfinlay.sld.cu/index.php/finlay/article/view/1317

[6] Ardila S, Echeverri OY, Guevara J, Espinosa E, Barrera LA. Tirosinemia de tipo I, aciertos y errores. Pediatría. 2014;47(3):55–9.

[7] Kaye CI; Committee on Genetics; Accurso F, La Franchi S, Lane PA, Hope N, Sonya P, Bradley S, Michele A LP. Newborn screening fact sheets. Pediatrics. 2006 Sep;118 (3):e934-63. 10.1542/peds.2006-1783 10.1542/peds.2006-1783 16950973

[8] Ibarra-González I, Ridaura-Sanz C, Fernández-Lainez C, Guillén-López S, Belmont-Martínez L, Vela-Amieva M. Hepatorenal Tyrosinemia in Mexico: a call to action. Adv Exp Med Biol. 2017;959:147–56. 10.1007/978-3-319-55780-9_14. 28755193 10.1007/978-3-319-55780-9_14

[9] Das AM. Clinical utility of nitisinone for the treatment of hereditary tyrosinemia type-1 (HT-1). Appl Clin Genet. 2017;24(10):43–8. 10.2147/TACG.S113310. 10.2147/TACG.S113310 PMC5533484 28769581

[10] Maiorana A, Malamisura M, Emma F, Boenzi S, Di Ciommo VM, Dionisi-Vici C. Early effect of NTBC on renal tubular dysfunction in hereditary tyrosinemia type 1. Mol Genet Metab. 2014;113(3):188–93. 10.1016/j.ymgme.2014.07.021. 25172236 10.1016/j.ymgme.2014.07.021

[11] Das AM, Mayorandan S, Janzen N. Diagnosing hepatorenal tyrosinaemia in europe: newborn mass screening versus selective screening. Adv Exp Med Biol. 2017;959:125–32. 10.1007/978-3-319-55780-9_11. 28755190 10.1007/978-3-319-55780-9_11

[12] Chakrapani A, Gissen P, McKiernan P. Disorders of Tyrosine Metabolism. In: Inborn Metabolic Diseases. Berlin: Springer; 2022. p. 355–67.

[13] Sniderman King L, Trahms C, Scott CR. Tyrosinemia Type I. In: Adam MP, Feldman J, Mirzaa GM, et al., eds. *GeneReviews®*. Seattle (WA): University of Washington, Seattle; May 25, 2017. PMID: 20301688 20301688

[14] Äärelä L, Hiltunen P, Soini T, et al. Type 1 tyrosinemia in Finland: a nationwide study. Orphanet J Rare Dis. 2020;15(1):281. 33046095 10.1186/s13023-020-01547-w PMC7549233

[15] Fernández-Lainez C, Ibarra-González I, Belmont-Martínez L, Monroy-Santoyo S, Guillén-López S, Vela-Amieva M. Tyrosinemia type I: clinical and biochemical analysis of patients in Mexico. Ann Hepatol. 2014;13(2):265–72. 24552869

[16] Zeybek AC, Kiykim E, Soyucen E, et al. Hereditary Tyrosinemia type 1 in Turkey: twenty year single-center experience. Pediatr Int. 2015;57(2):281–9. 10.1111/ped.12503. 25223216 10.1111/ped.12503

[17] McKiernan PJ, Preece MA, Chakrapani A. Outcome of children with hereditary tyrosinemia following newborn screening. Arch Dis Child. 2015;100(8):738–41. 10.1136/archdischild-2014-306886. 25564536 10.1136/archdischild-2014-306886

[18] Alvarez F, Atkinson S, Bouchard M, Brunel-Guitton C, Buhas D, Bussieres JF, et al. The Quebec NTBC study. Adv Exp Med Biol. 2017;959:187–95. 10.1007/978-3-319-55780-9_17. 28755196 10.1007/978-3-319-55780-9_17

[19] Giguère Y, Berthier MT. Newborn screening for hereditary Tyrosinemia Type I in Québec: update. Adv Exp Med Biol. 2017;959:139–46. 10.1007/978-3-319-55780-9_13. 28755192 10.1007/978-3-319-55780-9_13

[20] Van Ginkel WG, Rodenburg IL, Harding CO, Hollak CEM, Heiner-Fokkema MR, van Spronsen FJ. Long-term outcomes and practical considerations in the pharmacological management of Tyrosinemia Type 1. Paediatr Drugs. 2019;21(6):413–26. 10.1007/s40272-019-00364-4. 31667718 10.1007/s40272-019-00364-4 PMC6885500

[21] Kurihara K, Toyoda H, Amoano K, et al. Discontinuation of NTBC after liver transplantation in tyrosinemia type 1. Pediatr Int. 2018;60(11):1039–41. 10.1111/ped.13697. 30375135 10.1111/ped.13697

[22] Van Spronsen FJ, van Rijn M, Meyer U, Das AM. Dietary considerations in Tyrosinemia Type I. Adv Exp Med Biol. 2017;959:197–204. 10.1007/978-3-319-55780-9_18. 28755197 10.1007/978-3-319-55780-9_18

[23] Vockley J, Andersson HC, Antshel KM, et al. Phenylalanine hydroxylase deficiency: diagnosis and management guideline [published correction appears in Genet Med. 2014 Apr;16(4):356]. *Genet Med*. 2014;16(2):188–200. 10.1038/gim.2013.157 10.1038/gim.2013.157 24385074

[24] Van Ginkel WG, Pennings JP, van Spronsen FJ. Liver cancer in Tyrosinemia Type 1. Adv Exp Med Biol. 2017;959:101–9. 10.1007/978-3-319-55780-9_9. 28755188 10.1007/978-3-319-55780-9_9

[25] Nitisinone, Drugbank online. https://go.drugbank.com/drugs/DB00348, 2023 (accessed 25 April 2024).

[26] Nitisinona, Asociación española de pediatría. https://www.aeped.es/comite-medicamentos/pediamecum/nitisinona, 2020 (accessed 15 April 2024).

